# I overthink—Therefore I am not: An active inference account of altered sense of self and agency in depersonalisation disorder

**DOI:** 10.1016/j.concog.2022.103320

**Published:** 2022-05

**Authors:** Anna Ciaunica, Anil Seth, Jakub Limanowski, Casper Hesp, Karl J. Friston

**Affiliations:** a Centre for Philosophy of Science, University of Lisbon, Campo Grande, 1749-016 Lisbon, Portugal; b Institute of Philosophy, University of Porto, via Panoramica s/n 4150-564, Porto, Portugal; c Institute of Cognitive Neuroscience, University College London, WC1N 3AR London, UK; d Sackler Centre for Consciousness Science and School of Engineering and Informatics, University of Sussex, Brighton BN1 9QJ, UK; e Canadian Institute for Advanced Research (CIFAR) Program on Brain, Mind, and Consciousness, Toronto, Ontario, Canada; f Lifespan and Developmental Neuroscience, Faculty of Psychology, Technical University Dresden, 01069 Dresden, Germany; g Centre for Tactile Internet with Human-in-the-Loop CeTI – Cluster of Excellence, Technical University Dresden, 01062 Dresden, Germany; h Wellcome Centre for Human Neuroimaging, University College London, WC1N 3AR London, UK; i Department of Developmental Psychology, University of Amsterdam, Science Park 904, 1098 XH Amsterdam, Netherlands; j Amsterdam Brain and Cognition Centre, University of Amsterdam, Science Park 904, 1098 XH Amsterdam, Netherlands; k Institute for Advanced Study, University of Amsterdam, Oude Turfmarkt 147, 1012 GC Amsterdam, Netherlands

**Keywords:** Sense of self, Agency, Sensory attenuation, Active inference, Predictive processing, Depersonalisation

## Abstract

This paper considers the phenomenology of depersonalisation disorder, in relation to predictive processing and its associated pathophysiology. To do this, we first establish a few mechanistic tenets of predictive processing that are necessary to talk about phenomenal transparency, mental action, and self as subject. We briefly review the important role of ‘predicting precision’ and how this affords mental action and the loss of phenomenal transparency. We then turn to sensory attenuation and the phenomenal consequences of (pathophysiological) failures to attenuate or modulate sensory precision. We then consider this failure in the context of depersonalisation disorder. The key idea here is that depersonalisation disorder reflects the remarkable capacity to explain perceptual engagement with the world via the hypothesis that “I am an embodied perceiver, but I am not in control of my perception”. We suggest that individuals with depersonalisation may believe that ‘another agent’ is controlling their thoughts, perceptions or actions, while maintaining full insight that the ‘other agent’ *is* ‘me’ (the self). Finally, we rehearse the predictions of this formal analysis, with a special focus on the psychophysical and physiological abnormalities that may underwrite the phenomenology of depersonalisation**.**

## Highlights

• This paper examines potential mechanisms behind atypical sense of self and agency in Depersonalisation Disorder (DPD).
• Using Active Inference, we argue that aberrant somatosensory attenuation and precision weighting underlies DPD.
• Failures of somatosensory attenuation may disrupt the sense of agency and control over one’s perceptions and actions.
• In DPD, individuals may believe ‘another agent’ is controlling their thoughts, perceptions or actions.
• Unlike psychosis however, in DPD the individual maintains the full insight that the ‘other agent’ is ‘me’ (the self).

*“If I quieten my mind, I can still almost taste the colour and richness of life as I knew it before that point; the feeling of being your own agent of change, the feeling of plotting a course through life, and the sense of expectation”. (*Ciaunica & Charlton, 2018*).*

In daily life, our brains constantly receive a cascade of sensory information arising from both inside our bodies and our lived environment. For most of us, most of the time, these experiences seem to be tacitly accompanied by a *sense of self* – a sense of being an embodied agent within a world, among but distinct from others (Gallagher, 2000, Hohwy, 2007, Limanowski and Blankenburg, 2013, Seth, 2013, Zahavi, 2005). Everyday experience also seems to involve experiences *of agency*; namely, the feeling that I am in control of my own bodily actions, that I can leverage them to access and change the external world’ (Gallagher, 2000, Haggard, 2017).

Depersonalisation Disorder (DPD henceforth) is a condition characterised by profound alterations of one’s sense of self (Sierra & David, 2011), typically inducing distressing feelings of detachment or estrangement from one’s self (depersonalization) and/or one’s surroundings (derealisation) (DSM IV-TR fourth edition, text revision 2000)^1^.

Described already by Dugas in 1898 (Sierra & Berrios, 1998), these dramatic alterations are typically experienced as a ‘split’ or a ‘fracture’ between a detached ‘witness’ or an observing agent, and an observed acting self, body and world: “*When I’m having an episode of depersonalisation, it feels more like I’m watching myself doing things, but I’m not present for it. I’m witnessing myself… I ‘know’ I’m in control, but I’m not ‘feeling’ in control*” (Perkins, 2021: 44). Or: “*My perception felt as though it had been drawn back inside my head, almost as though I was looking at the world from the back of my head, and could see the back of my own eye sockets. (…) Essentially, it felt like there was a divorce or fracture between the world and me so that although my body was still in the world, my mind was only an observe*r” (Ciaunica, Charlton, & Farmer, 2020: 6).

The experience of a self-split can manifest as a self-detachment from (a) one’s body or body parts (low-level sensory and bodily aspects of the self); (b) one’s subjective feelings and emotions (affective aspects); and (c) one’s personal stories, memories, thoughts and future plans, often described by sufferers as a lack of a narrative or a ‘plot’ in one’s life (see Billon, 2016, Ciaunica and Charlton, 2018, Sierra, 2009, Simeon and Abugel, 2006). The overall impact of this ‘self-split’ makes people feel “not fully real” (Medford, 2012, Simeon and Abugel, 2006), and living on ‘automatic pilot’ (Perkins, 2021).

DPD often co-occurs in relation to traumatic events, severe stress and are associated with symptoms of anxiety, panic, and depression (Hunter et al., 2004, Lyssenko et al., 2018, Michal et al., 2016, Millman et al., 2021). The prevalence of DPD is around 1–2% in the general population (Hunter et al., 2004), with onset typically occurring before age 25. Strikingly, feelings of depersonalization are the third most common psychological symptom reported in the general population (after anxiety and low mood), especially among young people (Simeon, Knutelska, Nelson, & Guralnik, 2003). Yet its underlying neurocomputational mechanisms, and therefore, the link between biology and phenomenological markers remains poorly understood (see Seth, Suzuki, & Critchley, 2011 for an early attempt).

In this paper we propose a novel conceptual model of disrupted sense of selfhood in DPD through the lens of the Active Inference framework. We suggest that failures of somatosensory attenuation and consequent abnormal percepts—and beliefs—may underwrite aberrant self-model in DPD. This may lead to a disruption of agentive control over both perception (sensory attention) and action (sensory attenuation), triggering abnormal perceptions, and consequent aberrant beliefs of self-detachment.

Active Inference is a process theory that aims to capture the capacity of biological organisms such as human bodies to survive and thrive in volatile environments (Friston, FitzGerald, Rigoli, Schwartenbeck, & Pezzulo, 2017). It builds upon the Free Energy Principle (Friston, 2005), i.e., a formalisation and extension of the Schrödinger (1956) seminal idea that living organisms avoid entropy, by engaging in self-organisation with the goal of maintaining their homeostasis^2^ within optimal limits for survival (Clark, 2013, Hohwy, 2013). Within this framework, it has been proposed that the experience of a self is underwritten by an inferential hierarchy, whereby the self is an inferred model of endogenous, deeply hidden causes of behaviour (Apps and Tsakiris, 2014, Seth, 2013). Embodied agents act as self-modelling systems in the game of maximizing evidence for their self-model (Limanowski and Blankenburg, 2013, Limanowski and Friston, 2018, Limanowski and Friston, 2020).

Importantly however, the homeostatic balance of a self-organising system crucially depends on the system’s ability to engage with their environment, and cannot be achieved in isolation from it. Allostasis or anticipatory homeostatic control, is the process whereby agents select actions that (will most probably) bring about desired sensory outcomes, explicitly or implicitly modifying the causal structure of the environment, so as to guarantee the recurrence of desired outcomes in the future (Sterling, 2012).

The emphasis on the dynamic component of selfhood is key for our argument. While previous predictive processing approaches outlined the interoceptive (i.e., the perception of visceral signals, Craig, 2002, Seth, 2013) or affective facets of selfhood (Gerrans, 2018, Gerrans, 2020), our model takes into account the idea that the human body cannot achieve self-regulation without maintaining and engaging in active exchanges with its proximal environment (Ciaunica, Roepstorff, Fotopoulou, & Petreca, 2021a; De Jaegher & di Paolo, 2007). Crucially, unlike internal processing of automatic homeostatic regulation of visceral inputs—over which we have little control (e.g. we do not typically choose whether our heart will beat, or our bowels will make a noise)—the process of taking action in the world to secure survival is something that agents *can* control. Hence, the sense of self and agency in typical humans may be inextricably linked to the ability to feel in control over one’s bodily self, engaging in actions and movement ‘out there’ in the world.

Given that our bodily self is not a static and closed entity, but rather a dynamic and open system, literally constituted in relation to a proximal environment (Ciaunica and Fotopoulou, 2017, Ciaunica et al., 2021a) then somatosensory attenuation becomes a key part of the story of understanding how the self emerges as differentiated and yet related to its surroundings.

Indeed, in order to successfully prepare and engage in perception and action ‘out there” in the world, the human brain needs to be able to attenuate and process much self-related information ‘transparently’, in the background; e.g., bodily signals (Ciaunica et al., 2020, Limanowski and Friston, 2018). For example, in order to catch a ball, I need to be able to rely on fast and automatic somatosensory processing of my leg movements, i.e., ‘doing’ the running ‘without thinking’. The fact that I do not pay explicit attention to my leg movements while running does not mean that my brain is not keeping track of them. It simply means that it does the task so well, processing somatosensory information so smoothly, that I can afford to process it in the background, allowing me to successfully focus on salient events: catching the ball. It should be noted that, of course, not all kinds of self-related information are attenuated or ‘transparent’. On the contrary: the mere ability to make self-related representations ‘opaque’ is what may enable conceptual, narrative, or reflective self-experiences—it is in parts what makes human self-experience unique (cf. Metzinger, 2003). But this mechanism can go awry: If self-modelling is thus altered (i.e., disrupted somatosensory attenuation) this may lead to aberrant self-focus, i.e., the experience of a self-detachment or split between the ‘I’ who is doing the running, and the ‘I’ who is observing the running.

If our hypotheses are correct, then depersonalisation symptoms, although typically couched as “losing” one’s sense of self, may be the linked, on the contrary, to an inability to attenuate self-related inputs and hence to ‘forget’ the self in the background. Alterations in the ability to attenuate self-related information in order to optimally perceive, engage and act in the world may further lead to increased reflexivity or ‘hyper-reflexivity’ (Ciaunica et al., 2020, Fuchs, 2005, Sass and Parnas, 2003). In our example: over-attending to one’s leg movements while running may prompt people to detach themselves from the action, and see themselves from ‘above’. As we will see below, this bias towards self-related over-thinking and hyper-reflexivity, offsets diminished body-related processing. This hypothesis is consistent with subjective reports outlining feelings of being simultaneously trapped in one’s head (mind) and outside one’s body (disembodiment) (Ciaunica et al., 2020, Ciaunica et al., 2021a). Perhaps paradoxically, this imbalance may entail an abnormal elevation of higher-order self-related processing, rather than a ‘loss’ of the sense of self.

We unpack these hypotheses below as follows. In section 1 we briefly introduce the notions of somatosensory attenuation and transparent pre-reflective self. We then move in section 2 to present the active inference conceptual toolbox and its relation to the sense of self and the sense of agency over one’s actions. 3, 4 develop and motivate the claim that disrupted sensory attenuation and aberrant self-focus may trigger a ‘split’ in the sense of agentive control over one’s own perceptions and actions in DPD. We show how this is intimately related to the attentional augmentation and attenuation of sensory precision in the setting of active inference. We then connect these claims with the phenomenology of depersonalisation symptoms focusing on phenomenal transparency and qualitative experience. We conclude with a non-exhaustive list of testable predictions that our hypotheses imply. We also suggest some potential therapeutical implications of our approach that could usefully be explored, with the aim of improving the day-to-day life of people experiencing this distressing condition.

## 1 The importance of ‘Self-Attenuating’: somatosensory attenuation and the transparent pre-reflective self

When picking a ripe cherry from a tree to borrow an example from Limanowski and Friston (2020), we seem to be quite sensitive to the feel of the cherry, as we touch and grasp it. Yet we are almost insensitive to the feelings of our arm and eye movements while reaching the cherry, despite the fact these signals are essential in ensuring we successfully pick the ripe cherry, and not the green one next to it. Somatosensation (from ‘soma’ (body) + sensation) is an umbrella term referring to processing of tactile, thermic, proprioceptive, pleasure and pain signals through neural receptors in the skin. In our example, somatosensory information would include a set of signals about both the tactile perception of the cherry (softness, humidity, etc.) and the perception of one’s body in space and movement (position of fingers, kinaesthetic trajectory of the arm, etc.).

Seminal studies illustrated that we automatically anticipate the sensory effects of self-initiated actions (Post et al., 1994, Wolpert and Flanagan, 2001), which explains why people typically cannot tickle themselves (Blakemore et al., 1998). There is mounting psychophysiological and brain imaging evidence for this requisite attenuation of somatosensation during and prior to action is typically accompanied by a decrease in the primary somatosensory cortex responses (Bays et al., 2005, Palmer et al., 2016)^3^. These findings suggest that sensory attenuation plays a key role in action-initiation (Brown et al., 2013, Hughes et al., 2013; Parees et al., 2014, Zeller et al., 2015, Bhatt et al., 2016).

The close relationship between somatosensory attenuation, top-down precision control, and the flexible updating of body representation has been documented in scenarios like visuomotor adaptation and visuo-proprioceptive integration under conflict (see Limanowski, 2021, for a review). Importantly, somatosensory attenuation is key for building multisensory bodily-self representations, especially when sensory signals from multiple sensory modalities are conflicting (Limanowski and Friston, 2020, Paton et al., 2012, Zeller et al., 2015). It has been argued that attenuation of self-generated inputs gives rise to the feeling that one is in control of one’s own actions, or the sense of agency (Gallagher, 2000, Leptourgos and Corlett, 2020). Thus, it has been proposed that somatosensory attenuation (Haggard, 2017), and, generally, the comparison of predicted and actual somatosensory feedback underpins the distinction between oneself and the world (Fletcher and Frith, 2009, Frith et al., 2000); and specifically, self-other distinction (Haggard, 2017). While the relationship between sensory attenuation and the sense of agency is complex (Seth et al., 2011), it has been shown that agency over movements that generate sensation may be necessary for sensory attenuation to occur (De Santis et al., 2007, Gentsch and Schütz-Bosbach, 2011).

According to a longstanding phenomenological tradition, all our experiences imply a pre-reflective self or a ‘minimal self’ that makes my experiences immediately and tacitly given as *mine* (Fuchs, 2015, Merleau-Ponty, /1945, 1962, Zahavi, 2005). Importantly, pre-reflective self-consciousness should not be regarded as an extra layer added to the on-going experience; rather it essentially constitutes the very mode of being of *any* conscious experience (Sartre, 1943/1956). In other words, there cannot be an experience without a pre-reflective self at its very core.

Interestingly, this classic phenomenological approach echoes recent trends in mind and brain research stipulating that our perceptions, cognitions and actions are geared towards *self-*preservation (Azzalini et al., 2019, Barrett and Simmons, 2015, Ciaunica and Fotopoulou, 2017, Gallagher, 2000, Northoff and Panksepp, 2008, Panksepp, 1998, Seth and Tsakiris, 2018, Thompson, 2007, Wolpert and Flanagan, 2001). By maintaining and regulating the physiological needs and integrity of the organism (the human body), perceptual and sensory awareness at the most basic sensory level is inherently “selfish” (Ciaunica and Crucianelli, 2019, Seth and Tsakiris, 2018, Seth, 2021). A comprehensive review of this rich literature—on the different facets of the selfhood—lies beyond the scope of this paper (see Gallagher, 2013, Allen and Friston, 2016, Qin et al., 2020 for a review).

For our discussion here we retain the idea that a self-organising system such as the human body is most intimately acquainted with *self*-related signals. This means that the problem the brain has to solve is often “not which sensory evidence to *emphasise*, but which to *attenuate* (Limanowski & Friston, 2020:8, original italics) in order to optimally act in the world. This is because survival of an open and vulnerable self-organising system—such as the human body—depends on the ability to engage in homeostatic and allostatic regulation, via active exchange with one’s surroundings (Allen and Friston, 2016, Sterling, 2012).

This means that the most basic parts of the self-model are unique, in the sense that they are “necessarily transparent” (Limanowski & Friston, 2018). Transparency is an interesting and peculiar property of our experiences which has been theoretically spelled out in different ways by different theorists (Ciaunica et al., 2020, Fuchs, 2005; Metzinger, 2003, Moore, 1903, Tye, 1999) and a detailed review of these accounts^4^ lies beyond the scope of this paper. In a nutshell, transparency can be intuitively grasped via the so-called ‘window’ metaphor. For example: a perfectly clear and transparent window glass or sliding door can give us the illusion of an unmediated access to a landscape, say. The landscape seems present and reachable paradoxically because the window’s glass is transparent, invisible and taken for granted: it is there without us being aware that it is there. However, in some cases, as we will see later, one *can* become aware of the existence of the invisible and mediating transparency of the window itself so to speak. Indeed, suppose there is a crack in the pane of glass. Two important observations emerge here: a) first, we become aware of the cracked window itself as a *visible* observable entity: we realize that there was something there without us being aware of its presence in the first place. b) Second, while the cracks in the window’s glass make the latter visible, they also make our access to the landscape more ‘opaque’ or ‘mediated’. We may still perceive the outer landscape through the cracked window, but its clarity is hindered. Now we are aware that something stands in the way, and disrupts our full immersion into the reality of the landscape. In sum, two key points are to be retained: (a) the property of transparency enables the subjective feeling that we are in immediate contact with our self and the world, and (b) that consequently both self and world are felt as being real and present.

Importantly, this idea can be applied to self-modelling as well: “just as a transparent world-model grants the experience of being in immediate touch with the world, a transparent phenomenal self-model…affords the experience of being in immediate relation to a self” (Limanowski and Friston, 2018, Metzinger, 2003). We call this basic default-mode of self-processing ‘transparent self-modelling’, as developed below. We postulate that this may correspond to the pre-reflective self as defined by the phenomenological tradition (Fuchs, 2005, Sartre, 1943).

In what follows, we rehearse the key concepts of self- and world-modelling, ‘precision’ and ‘precision weighting’ and review suggestions that aberrant precision control may disrupt the ability to infer accurate self- and world models in various conditions. We then turn to the case of DPD (section 3) and suggest that aberrant precision estimation—biased towards ego-centric priors—means that the luxury to engage in transparent self-modelling is denied. We turn to this discussion now.

## 2 Prediction (im)precision: altered self-models through an active inference lens

Active inference draws on von Helmholtz (2005) seminal idea the brain constructs a mental representation of sensory inputs via perceptual inference, whereby prior percepts automatically shape the percept that is generated by the incoming sensory information. This idea has inspired the modern approach of perception as predictive processing (Clark, 2013, Friston, 2005, Hohwy, 2013, Knill and Pouget, 2004, Rao and Ballard, 1999). As we saw earlier, the most ‘newsworthy’ and pervasive information that our brain receives and needs to process optimally is *self*-related information.

Within this framework, self- and world-modelling is organised in a dynamic and hierarchical fashion. Prior beliefs^5^ about the self and world generate predictions that are conveyed by the top-down (backward) connections to lower hierarchical levels. Bottom-up (forward connections) return prediction errors to update prior beliefs — into posterior beliefs — until prediction errors are explained away by ensuing belief updating. In a hierarchical setting, this enables sensory input at the lowest level of the hierarchy to be assimilated through prediction. Posterior beliefs are hypotheses concerning the causes of sensory input at any hierarchical level that therefore rest on (1) *prior beliefs* about the self and world and (2) *current sensory evidence* gathered from a volatile and ever-changing environment.

In updating the self- and world-models in order to optimally adapt within a dynamic and potentially threatening world, much depends on the ‘*precision*’ of the prior prediction and the sensory prediction error induced by sensations. Prior beliefs and sensory data are represented as probability distributions with (a) mean value (expectations) and (b) precision (inverse variance). Now, if prediction errors are based on precise sensory data and relatively imprecise prior beliefs, the mean of the posterior will be closer to the mean of the sensory data. By contrast, if sensory information is deemed imprecise, posterior beliefs will be much closer to prior beliefs. This means that predictions of precision—or predictions of predictability—can have a profound effect on hierarchical belief updating in the brain.

Because of the inherent volatility of the incoming sensory information from both inside and outside one’s body, the process of updating self- and world-modelling needs to be flexible enough to allow the system to assess which input is more ‘trustworthy’. Now, in updating one’s self- and world-models, much depends on the relative precision of expectations versus sensory evidence. Fine-tuning the weighting of prior beliefs and sensory evidence is often called *precision weighting*, which translates to selectively attending to (or ignoring) particular sources of evidence. Note that precision control has a fundamental role in the construction of self-representations. The challenge that an adaptive living organism faces is to ‘decide on the fly’ whether the weight of the balance—the ‘gain’ of the updating process— is afforded to the (a) sensory evidence from various modalities or (b) to the prior beliefs (or expectations) that have to explain the sensory inputs.

It is important to note that the system is also trying to predict precision. Precision weighting has been linked to *attention* as the process of affording precision to (i.e., placing confidence in) certain aspects of the sensorium (Feldman and Friston, 2010, Hohwy, 2021). Precision optimisation is a mechanism that allocates ‘weight’ or ‘gain’ either to sensory input or prediction errors higher in the hierarchy. Given that precision-weighting works as a kind of ‘searchlight’, this makes it a “promising candidate for the mechanism for *attention*” (Hohwy, 2021:9, original italics). Indeed, the precision of sensory data and prior beliefs is not fixed: it can be optimized by attention to best reflect uncertainty about their contribution in any given context. In short, attention may be underpinned by (context sensitive) mechanisms assigning greater or lesser precision to prediction errors at various levels of the hierarchical processing.

The other side of this coin is the attenuation of sensory precision that undergirds sensory attenuation. This selective *dis-attention* may be a crucial faculty that enables us to ignore sensory evidence that we have not acted, when we think we are acting. This transient suspension of attention to the consequences of action enables reflexes to realise our predicted (i.e., intended) actions in both motor and autonomic domains. In short, precision optimisation plays a key role in action-initiation**.** In acting, the agent simultaneously generates a prediction of the sensory input expected to result from the intended movement, and ‘self-fulfils’ this prediction by *doing* the movement. This involves successful suppression of the prediction errors that would otherwise subvert movement: namely, provide irrefutable evidence that “I am not moving”, despite my prior belief or intention to move (Adams et al., 2013, Brown et al., 2013, Friston et al., 2010, Seth and Friston, 2016).

What happens if the process of precision estimation itself gets disrupted? For example, what happens if the precision weighting gets ‘stuck’ and tilts systematically towards one of the two branches (i.e., bottom-up sensory evidence or top-down prior belief) at various hierarchical levels? Such aberrant precision control precludes the flexibility afforded by an adaptive modulation of ascending prediction errors, typically associated with optimal synaptic gain control and successful belief updating. More importantly, as we will see shortly, disruptions at the level of precision prediction means that we no longer have the luxury to ‘ignore’ or dis-attend certain levels of the hierarchical processing of the self-model (i.e., we lose the ability to see the sensorium for what it is).

The idea that aberrant precision control disrupts the ability to infer accurate self- and world models—thereby triggering abnormal perceptions and beliefs—has been linked to various conditions such as depressions, autism and apathy(see Heinz and Schlagenhauf, 2010, Van de Cruys et al., 2013, Corlett and Fletcher, 2015, Friston, 2017, Sterzer et al., 2018, Smith et al., 2019, Stephan et al., 2016, Hezemans et al., 2020).

Crucially, disrupted precision balance has been related to disorders of selfhood such as psychosis and schizophrenia. For example, there are current debates regarding psychosis as linked to an increased or decreased precision in the encoding of prior beliefs relative to the sensory evidence (c.f., a failure of sensory attenuation), thereby engendering maladaptive inferences (e.g., misattribution of one’s voice to an ‘other’) (Corlett et al., 2009, Fletcher and Frith, 2009, Sterzer et al., 2018). While further work is needed to disentangle these aspects, one may speculate that these approaches are compatible insofar as different experimental designs tackle distinct levels of the hierarchical processing. For example, if one endorses a developmental perspective in understanding the “first priors” (Ciaunica, Constant, Preissl, & Fotopoulou, 2021b), then one may argue that different senses (e.g., tactile versus visual inputs) may have preferential access to higher levels of the hierarchy. For example, affiliative touch may be afforded more precision than visual afferents, and this may trigger distinct precision weighting at further levels of hierarchical processing.

A significant body of work found that sensory attenuation is also reduced in schizophrenia (Blakemore et al., 2000; Brown et al., 2013, Fletcher and Frith, 2009, Shergill et al., 2005). More specifically, this deficit transpires to be a failure of sensory attenuation that can be attributed to aberrant precision control that confounds inference about the causes of self-generated sensations (Brown et al., 2013; Oestreich, Mifsud et al., 2015; Parees et al., 2014). Failures of sensory attenuation mean that the percepts of schizophrenic people can be less malleable and more veridical than controls; hence, their characteristic resistance to illusory phenomena. A key symptom of schizophrenia is aberrant perception of agency (Frith, 2005) with the delusion that one’s actions are controlled by others. This has been linked to deficits in the patients’ generative model (Frith, 2005), and an inability to retune their model to elude cognitive deficits and psychiatric symptoms (Kilteni, Houborg, & Ehrsson, 2019).

In the remainder of this paper, we suggest that aberrant (pathophysiological) precision control underwrites a failure of somatosensory attenuation in DPD, which precludes the processing of self-generated sensations ‘transparently’ in the background. These disruptions may lead to feelings of ‘overthinking’, hyper-reflexivity, opacity and consequent lack of presence in the world or ‘realness’ of one’s experiences. Disconnection from one’s body may also explain the sensations of being unreal, and navigating through the world surrounded by a ‘pane of glass’, ‘experiential airbag’ or ‘opaque veil’ interposed between one’s self, body and the world (Ciaunica and Charlton, 2018, Sierra, 2009, Simeon and Abugel, 2006).

## 3 Over-inferencing the self – from aberrant self-modelling to agentive self-split

Previous work used the predictive processing framework to link DPD symptoms to pathologically imprecise interoceptive signalling – the perception of visceral signals (Craig, 2002) – which consequently fails to update higher-level beliefs and thus perpetuates a sense of ‘unrealness’ (Seth et al., 2011). More recently, impaired self-related affective processing has been advocated as core feature od DPD symptoms (Gerrans, 2018, Gerrans, 2020).

Our proposal takes this line of work a step further and suggests that altered sense of self in DDD may be linked to disrupted self-modelling in relation to somatosensory attenuation of one’s bodily self in action. We have seen above that sensory attenuation may underwrite a feeling that one is in control of one’s perceptions and actions (i.e., feelings of agency): ‘I infer that I am the agenta of these sensed actions’ because any evidence to the contrary is attenuated. To put it differently: in order to successfully engage in actions out there in the world, the brain needs to attenuate *self*-related information. Because self-related and self-generated inputs are so pervasive, in typical cases, they are not attentively processed, as highly predictable and hence ‘boring’ information. This relates nicely with previous work on why people typically cannot tickle themselves: self-generated action and perception is anticipated. The key idea is that in order to be able to focus on newsworthy information (e.g., there is an edible cherry within arm’s reach), the brain can afford to process self-related information transparently in the background (i.e., self-attenuation).

Now, let us imagine that the brain has to deal with high levels of imprecision or unpredictability on a constant basis. Consequently, self-related information is often taking the ‘headlines’ of the newsworthy inputs, so to speak, and thus self-attenuation is impaired. For example, let us imagine you need to move in a radically new culture, where the levels of new information are significantly higher than before, and you have to constantly update your self- and world-models. Or you need to face constant stress related to a demanding work or study environment. Or you need to process a highly unexpected and traumatic event: you went for a ride with your partner, but you had a car crash and your partner died. Or you went to a party to have fun, you took some recreational drugs, but the changes in your perception are so radical that your brain is overwhelmed, and you experience panic attacks instead. All these experiences, although triggered by different type of events, have in common the fact that they involve high levels of uncertainty and unpredictability processing (e.g. short and intense, or long and systematic), and hence a perceived loss of control over one’s bodily self and actions.

Yet, as we saw earlier, one key idea within the active inference framework is that the brain’s main task is to keep track of self-related information in order to ensure bodily survival. Consequently, if the latter is considered to be in danger, then the brain will allocate more computational resources to harvest this highly newsworthy information: endangered self-preservation. In typical cases, if I need to do a new and delicate task (e.g., opening a bottle of champagne for the first time), I may pay extra attention to my bodily actions and ensure I do not hurt my partner with the cork. But what if every single action I do in the world is perceived by my brain as new and potentially dangerous? What if I cannot afford to “forget” about my self, but rather I need to keep track of it constantly?

Here, we propose that high levels of uncertainty and unpredictability may result in feelings of “losing control” over one’s bodily self and actions, triggering compensatory sub-optimal mechanisms of over-control of one’s self and bodily actions. Paradoxically however, as we saw earlier, sense of agency crucially depends on the ability to leave the self in the background (i.e., sensory attenuation).

Aberrant precision control—of ascending prediction errors—will lead the system, as a side-effect, to ‘over-attend’ to its own self-models. Going back to our initial metaphor: if there is a loss of the window’s transparency, opacity will ensue and sensations are ‘experienced’. Considerable computational resources are mobilised during these situations, which shift the self-model from the ‘transparent’ invisible background to the visible and ‘opaque’ upfront. The resulting self-model in this case will most likely infer altered self-experiences and consequent hyper-reflexivity. At the experiential level, this process may correspond to what phenomenologists call ‘self-objectification’ (Fuchs, 2005): by allocating extra resources to the processing of its own model, the self treats itself as an object to be controlled and ‘grasped’—very much like the cherry in the previous example.

Crucially for our thesis, increasing sensory precision entails a reduction of sensory attenuation, which is especially prescient when modelling oneself. As Limanowski & Friston put the point: “The temporary attenuation of the precision of sensory “self-evidence” – which is necessary to entertain an alternative (and yet counterfactual, c.f., Seth 2014) hypothesis about myself – is effectively a form of “self-attenuation” (2020: 10).

It is important to retain that the fine-grained predictive model of the moment-to-moment changes in sensory input—that are expected on the basis of one’s own planned movement— usually attenuate the sensory consequences of action. This enables us to ignore the fact that we are not moving prior to the execution of a movement. If this sensory attenuation fails, the inability to ignore the sensory consequences of self-made acts may result in a false attribution of agency: i.e., ‘you did that, not me’ (Blanke et al., 2015, Faivre et al., 2020, Salomon et al., 2020, Synofzik et al., 2010, Voss et al., 2010). Thus, the sensory consequences of one’s own actions generate unattenuated prediction errors that are read as evidence by the brain that this was not one’s own agentic movement (Sterzer et al., 2018). Indeed, as we saw above, a common feature of disorders of selfhood—such as psychosis and schizophrenia—is a perceived loss of agency: e.g., one’s actions and thoughts are experienced as controlled by external agents, the so-called passivity phenomena (Waters & Badcock, 2010). For example, it has been argued that the fine-grained predictive model of the moment-to-moment changes in sensory input that are expected on the basis of one’s own planned movement is relatively imprecise (Synofzik et al., 2010, Voss et al., 2010). Thus, the sensory consequences of one’s own actions are associated with an unusually high prediction errors at this level, suggesting that this was not one’s own agentic movement (Sterzer et al., 2018). This hypothesis seems supported by several lines of evidence. For example, psychosis has been associated with a greater resistance to visual illusions (which rely on prior beliefs for their effects), a failure to attenuate sensory consequences of self-generated actions, impaired smooth visual pursuit of a moving target, but improved tracking of unpredictable changes in target motion, a decreased influence of stimulus predictability on brain responses [e.g., N400, P300, mismatch negativity; and a loss of corticothalamic connectivity (see Adams et al., 2013, Notredame et al., 2014 for reviews).^6^

Our proposal is that failures of sensory attenuation in DPD may therefore disrupt the sense of agency over the perceived consequences of action. Indeed, if significant deviation from the predicted sensory consequences of my actions occurs—or sensory evidence is unattenuated before the consequences are sensed—then the most plausible explanation for the system may be that ‘I am not in control of my actions’, but ‘some other agent is’.

If this is so, then DPD may reflect the remarkable capacity to explain perceptual and active engagement with the world with two mutually exclusive but equally plausible hypotheses. (1) First, a hypothesis that the best explanation for all the evidence at hand is that “I am an embodied perceiver, and I am in control of my perceptual processing”. (2) The alternative hypothesis is that “I am an embodied perceiver, but I am not in control of my perceptual processing”. These permit a dissociation between controlled perception and the agency of that control.

By treating self- and world-modelling itself as a process being controlled by a ‘self’, the latter is perceived simultaneously as being (a) an ‘other’ external agent; and (b) *my* internal self. An important corollary of having alternative self-models in play is that one immediately introduces uncertainty about which model is fit for purpose in explaining the sensory data. The capacity to entertain uncertainty about ‘what sort of self I am’, may also explain the stress and negative effective valence associated with depersonalisation. This follows from the fact that all the available evidence suggests that negatively valanced experiences and stress can be traced back to a loss of confidence or certainty in representations of how to engage actively with the world (Badcock et al., 2017, Peters et al., 2017). In one sense, perhaps the most fundamental sort of anxiety and stress would be associated with the existential uncertainty about “the sort of self that I am”.

Our proposal in consistent with previous work highlighting a close relationship between anxiety and DPD (Baker et al., 2003; Hunter et al., 2003; Michal et al., 2016, Simeon et al., 2003) although this link is complex and needs further investigation. For example, Sierra and colleagues assessed levels of anxiety and depersonalization in 291 consecutive DPD cases. 'High' and 'low' depersonalization groups, were compared according to anxiety severity. They reported that a low but significant association between depersonalization and anxiety was only apparent in those patients with low intensity depersonalization, but not in those with severe depersonalization. A more recent study (Millman et al., 2021) assessed the extent to which symptom heterogeneity in DPD reflects the presence of five discrete latent classes (low-, moderate- and high DPD severity, High depersonalisation and High dissociation (Brown, 2006). The authors found that anxiety was not a strong indicator of class differences within their sample. Specifically, all five classes were relatively comparable in anxiety scores with the exception of High severity class, which showed the most severe score. As the authors note, their findings are at odds with the study by Sierra, Medford, Wyatt, and David (2012) mentioned above. Further work needs to disentangle which aspects of DPD are intrinsically related to anxiety.

In the last section, we explore potential links between the mechanisms subserving altered somatosensory attenuation in DPD and its associated phenomenology. We will then conclude with some testable hypotheses that our model entails, and future directions.

## 4 The split ‘I’: linking mechanisms and phenomenology of altered somatosensory attenuation in depersonalisation

Hitherto, we have seen that dealing with high levels of uncertainty may alter the brain’s ability to attenuate self-related information, which in turn may lead to a compensatory emphasis on metacognitive, higher-level modes of self-awareness or ‘hyper-reflexivity’ (Ciaunica et al., 2020, Fuchs, 2005, Sass and Parnas, 2003). If one feels that things are ‘out of control’, the natural reaction is to try to regain control, by allocating extra perceptual and computational resources to ensure self-preservation (i.e. enhanced attention to the ‘I’). This compensatory mechanism constitutes an optimal response to a potential threat, as long as it remains transient (e.g., “I pay extra attention while I open a bottle of champagne to ensure I will not harm myself and others”). However, prolonged allocation of extra perceptual and computational resources to the self—to the detriment of relating to the world and others—may result in feelings of being detached; not only from the world and others, but more importantly from one’s self as well. This paradox may be explained by the fact that our sense of self is an open-ended process, constantly fuelled and transformed via dynamic exchanges with the physical and prosocial world. Impoverished exchanges with the environment and feelings of being ‘cut off’ from the outer world may lead not only to overly ruminative inner workings, but also to feelings of being ‘cut off’ from oneself (Ciaunica et al., 2021a).

At the experiential level, this process may correspond to what phenomenologists call ‘self-objectification’ accompanied by a loss of transparency of one’s basic pre-reflective sense of self (Fuchs, 2005, Sass and Parnas, 2003). One could see this as a loss of phenomenal transparency, not concerning only the contents of perception, but regarding the normally transparent control of sensory attenuation and ensuing attention. The ‘I’ becomes overly self-aware and ‘stands in the way’—so to speak—between the agent and its own bodily self and actions. Such an overt metacognitive self-awareness will contribute to a ‘split’ between the ‘I’ as a subject of an experience and the ‘me’ as an object of my awareness: “*I feel sometimes that it’s not me who sees the things I see in a way. I know it’s me, but it feels like my consciousness is somewhere else, as if I’m not experiencing the things I see*” (Værnes, Røssberg, & Møller, 2018: 202).

Consistent with this view, an enhanced tendency towards obsessional self-checking of one’s internal states has been consistently reported by DPD patients (Ciaunica et al., 2022a, Hunter et al., 2003, Hunter et al., 2004, Medford et al., 2005, Simeon and Abugel, 2006). ‘How do I feel now?’, ‘Who am I’, ‘Why do I feel the way I feel?’: these existential, philosophical questions on the nature of the ‘mind’, ‘self’, ‘existence’ and ‘reality’ are quite common in DPD, who are often drawn to rumination and over-intellectualization of their inner workings. Patients’ attention is monopolized by the strangeness of one’s internal states, triggering simultaneously inner turmoil and non-responsiveness to external world (Hunter et al., 2003).

Here we hypothesize that alterations of the ability to attenuate self-related sensory processing are key to the pathophysiology of DPD. Our proposal is also consistent with previous work outlining that DPD may be related to a form of *pathological attentional bias* and atypical multisensory integration of self-related information, in which *aberrant salience* is misattributed either to internal (interoceptive) bodily signals or external (exteroceptive) information (Hunter et al., 2003, Medford, 2012, Sass et al., 2013).

To put it simply, depersonalization may be seen as a type of ‘passivity phenomenon’: if my perceived bodily sensations depart from my expectations all the time, I could start believing that they are not mine: c.f., delusions of control. However, I were able to downregulate my confidence in my own expectations (i.e., a form of metacognition), I could maintain a higher-level belief that I am still in control of my sensations, even though it does not feel like that: *I feel like a robot, like I am listening to someone else talking, like I am looking at myself from the outside, but it is not another voice or body - it is mine, it is me, it just doesn’t feel like it.”* (Baker et al., 2003). Interestingly, these observations are in line with previous research showing that passivity symptoms can be linked to an altered sense of agency in schizophrenia patients. For example, a stronger self-attribution bias—individuals’ misperception of a limb as being their own (Farrer et al., 2003, Farrer et al., 2003, Tsakiris et al., 2005)—has been found in schizophrenia (Daprati et al., 1997, Franck et al., 2001). Crucially however, while there is a significant overlap of dissociative symptoms between depersonalization and psychosis (Sass et al., 2013), reality testing remains intact in DPD.

**Fig. 1:**
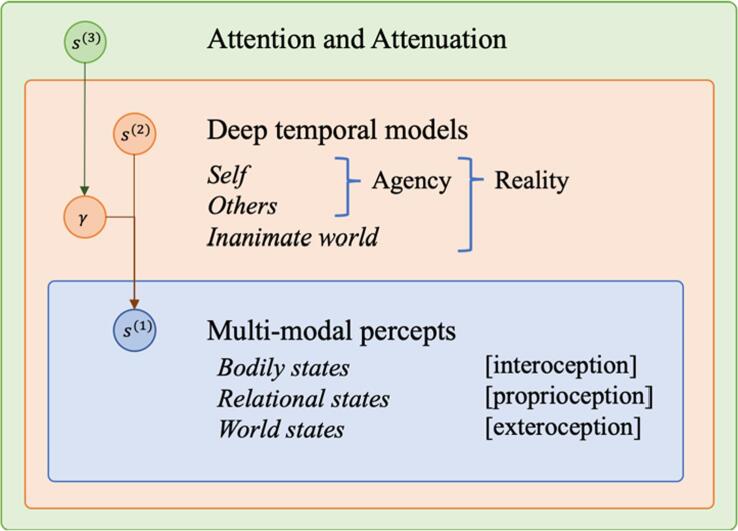
This simplified generative model illustrates the inferential process of explaining *multi-modal percepts* ($s^{\left(1\right)}$; blue) in terms of *deep temporal models* ($s^{\left(2\right)};$ orange) for which the precisions γ are set by higher-level states of *attention and attenuation* ($s^{\left(3\right)}$; green). Self and Others are models of agency (or intuitive psychology), which often exhibit large degrees of overlap (Friston & Frith, 2015), while one’s model of the inanimate world is governed by intuitive physics (see Ullman, Spelke, Battaglia, & Tenenbaum, 2017). The highest level performs Bayesian model selection to guide inferences about which combination of the deep temporal models (Friston et al., 2017) provides the best explanation of the multi-modal percepts of one’s body (interoception; Seth et al. 2011; Allen, Levy, Parr, & Friston, 2019), world (exteroception; Parr, Corcoran, Friston, & Hohwy, 2019). For a computational implementation of Bayesian filtering with multiple internal models, see the work by Isomura, Parr, and Friston (2019). Such models are temporally deep in the sense that they involve Bayesian inference on multiple time scales (Hesp et al., 2020, Ramstead et al., 2018): observations in ‘real-time’ inform beliefs about lower-level parameters (intermediate time scales), which in turn allow for updating beliefs about higher-level parameters (successively larger time scales). (For interpretation of the references to colour in this figure legend, the reader is referred to the web version of this article.)

The specific neural and computational mechanism behind a failure of sensory attenuation in DPD is currently an open question. Here, we speculate that a core mechanism involves imbalanced precision weighting towards self-priors, leading to the inability to flexibly update the self- and world-models as new information is accumulated. These disruptions may be linked with an aberrant higher precision allocated to internal milieu (e.g., interoceptive) signals, resulting in enhanced self-focus and inability to attenuate self-induced stimulation and actions. A detailed mathematical description of aberrant self-modelling in DPD is beyond the scope of this paper and will explored in future work (Authors et al., in prep. See Fig. 1).

Our model builds upon the premise that adaptive behaviour depends on keeping an optimal balance between top-down and bottom-up driven attention over self- and world-induced sensory signals. The hypothesis that DPD seems to be imbalanced towards bottom-up modes is supported by evidence suggesting a stronger impact of exogenous attention and underlying neuronal abnormalities in these pathways in DPD (Corbetta and Shulman, 2002, Simeon et al., 2000). Empirical support for this disrupted bodily sensory processing comes from studies that demonstrate disrupted physiological responses in patients with DPD, compared to healthy participants (Dewe et al., 2018, Owens et al., 2015, Sierra et al., 2002). Other studies found altered somatosensory and attentional functioning at early processing stages in depersonalisation (Adler, Schabinger, Michal, Beutel, & Gillmeister, 2016) but not in anxiety- and depression-matched patients (Schabinger et al., 2018). DPD has also been linked to disrupted activity in neuronal regions underlying somatic processing (Lemche et al., 2013, Medford et al., /2012, 2016) and the vestibular system (Jáuregui Renaud, 2015), which is responsible for providing information about the body’s position in space (Ferrè and Haggard, 2016, Ferrè et al., 2014).

The core mechanistic pathophysiology of aberrant precision weighting underlies a number of specific hypotheses connecting this mechanism to the phenomenology of DPD. First, a failure to attenuate interoceptive and exteroceptive self-related sensory signals would lead to an increase in interoceptive sensitivity and accuracy, to the detriment of a balanced and optimal coupling between signals coming from inside and outside one’s body, which is considered to be a key component of bodily self-consciousness (Park & Blanke, 2019). This may also transcend into the exteroceptive domain. For example, we anticipate that DPD correlates positively with over-sensitivity to visual and auditory sensory self-related signals (e.g., seeing one’s face in a mirror, or hearing one’s voice on a recorder). These alterations may trigger sub-optimal behaviours, which may lead to inhibitory, uncanny effects. As one patient with DPD puts it: “*The loss of the sense of self is a constantly perturbing experience. Looking at my face in the mirror feels like an uncomfortable staring contest with a total stranger*” (Perkins, 2021:41). Intriguingly, these sensations of self-estrangement seem to be closely linked with feelings of disembodiment and detachment from the reality: “*I look in the mirror and it doesn’t feel like myself I’m looking at. It’s like I’m floating, not actually experiencing the world, and slowly fading away into nothing. It’s like I’m on autopilot in somebody’s else body*” (Perkins, 2021:198) (see also Simeon and Abugel, 2006, Sierra, 2009).

Second, we predict that enhancing bodily self-focus via self-face observation, for example will improve interoceptive accuracy in low DP but not in high DP participants (due to a disrupted ability in integrating signals arising from outside and inside their bodies). For example, a study by Ainley, Maister, Brokfeld, Farmer, and Tsakiris (2013) explored the effect of enhancing healthy peoples’ attention to: (a) low-level, perceptual and bodily aspects of the self (gazing at one’s face in a mirror) as well as (b) high-level, cognitive and narrative aspects of the self (looking at autobiographical words evoking personal memories and traits). Their results suggest that both (a) and (b) conditions can improve interoceptive awareness (i.e. accuracy in perceiving one’s heartbeats), specifically in individuals who have initially low interoceptive awareness. Our model predicts that enhancing narrative (cognitive) self-focus via observing self-related (autobiographical) words will decrease interoceptive awareness in high DP participants. This is due to the fact that DP people’s tendency to mentally overscrutinize their own inner life negatively impacts their bodily awareness and the capacity to ‘listen’ their bodies.

Our model also predicts that dynamic sensory feedback (e.g. listening the sound of one’s footsteps) may increase not only (a) the feeling of being in control of one’s body and actions, but also (b) the malleability of one’s sense of self, allowing DPD people to feel less ‘stuck’ in one’s head (mind), and ‘putting them back’ into their bodies. Recent studies have shown that altering in real-time the sound of the footsteps produced by people as they walk, to make these sounds consistent with those produced by a lighter or heavier body, can result in changes in body representations (Tajadura-Jiménez, Basia, & Deroy, 2015). This suggests that our bodily self is not a fixed image that we see from the ‘outside’, but rather a flexible, fluid and dynamic representation, constantly updated through something as trivial as the sound of own footsteps.

Also, given that aberrant somatosensory attenuation may lead to hyper-reflexivity and over-intellectualisation of one’s experiences, we predict that people with depersonalisation will report to feel closer to their ‘former’ or ‘normal’ self during their dreams (Gillmeister & Ciaunica, in prep). This is because in their awake life, over-mentalization fuels abnormally their self-models, preventing them to feel fully in touch or immersed in their daily lives. By contrast, this hyper-reflexivity is diminished during the non-awake life, which should lead to an increase of their transparent self-modelling and consequent feelings of being again in touch with their ‘former’ self.

In fact, preliminary data from our ongoing studies suggest that people with high-levels of depersonalisation experiences will show a modulation of the magnitude of self-prioritization of self-associated bodily (avatar faces) versus abstract stimuli (geometrical shapes) in the sequential matching task (Wozniak, McEllin, Hohwy, & Ciaunica, 2022). For example, several studies demonstrate that self-related stimuli (e.g., one’s face or name) are processed faster and more accurately than others’ names and faces (Alexopoulos et al., 2012, Bortolon and Raffard, 2018, Woźniak and Hohwy, 2020). Specifically, our preliminary results indicate that depersonalisation individuals show less of the self-prioritization effect than the typical controls in the self-associated *bodily* task (avatar faces). However, they perform equally as the typical controls in the self-associated *abstract* task (geometrical shapes) (Wozniak et al., 2022). This is due to the fact that processing and integrations of bodily-related signals is impaired in DPD, while the processing of mentalistic (abstract) self-related processing is enhanced (hyper reflexivity). Along the same lines, the authors also found that activities involving high level and abstract cognitive abilities (e.g., participating in e-meetings via digital platforms such as Zoom, Teams, playing computer game, etc.) are positively correlated with higher levels of depersonalisation. By contrast, more basic and ‘humble’, body- and movement-based abilities (e.g., manual workings, physical exercise, etc.) will be positively correlated with low levels of depersonalisation (Ciaunica et al., 2022b). Again, experimental tests of these ideas will have to be carefully assessed in order to exclude potential confounding effects of demand characteristics (Lush, Botan, & Scott, 2020).

Finally, one would anticipate that people with depersonalisation disorder should show failures of sensory attenuation. In other words, they will show reduced psychophysical and electrophysiological response to stimuli caused by self and other, in relation to typical controls. They will also show a different pattern of responsiveness regarding affective touch. From previous literature, gently stroking the skin at a medium velocity (3–10 m/s, Löken et al., 2009) activates a special subclass of receptors that code for pleasant touch. We predict that people with high levels of depersonalisation experiences will rate affective touch experiences as significantly less pleasant and less vivid than the typical controls (Ciaunica et al., 2021c, Löffler et al., 2022). As above, demand characteristics would again have to be controlled for, or ruled out, in experimental tests (Lush et al., 2020).

Crucially, unlike in the case of psychosis, in DPD the meta-awareness state ‘It is I who experiences this split” remains intact, which may explain why the depersonalisation patients don’t ‘buy’ into the self-detachment story itself, and remain dramatically aware of the subjective nature of the experienced split (i.e., reality testing intact). This intact awareness may explain why “the distressing complaints of patients with depersonalization do not seem to be accompanied by observable changes in behavior” (Sierra, 2009:132). It is crucial however to better understand the experience of depersonalization because, as one person with DPD strikingly puts it “*a disorder that makes you feel invisible, is invisible in society*” (Perkins, 2021:193).

## 5 Conclusion and outlook

In this paper, we have examined some potential mechanisms behind an atypical sense of self and sense of agency in Depersonalisation Disorder (DPD), a condition in which people experience a ‘split’ or detachment from oneself, one’s body and the world.

We used the Active Inference framework to argue that atypical self-modelling—underpinned by aberrant precision control and sub-optimal sensory attenuation—disrupts the *transparency* of basic, pre-reflective forms of self-awareness in Depersonalisation Disorder.

If our argument is correct, then future research could usefully assess whether active multisensory engagements with the world and others via body-based, dynamic proximal (tactile and olfactory) interactions enhance the sense of self, realness and presence in people with DPD. We hypothesise that close and dynamic physical and synchronous interactions with their environment will make DPD people feel more present in their bodies, and less ‘trapped’ in their minds. This is because, paradoxically, in order to get closer to oneself, one needs to feel safe enough to be able to ‘forget’ oneself, and to focus instead on (inter)acting with the world and others, via proximal multisensory interactions (Ciaunica et al., 2021a).

The emphasis thus needs to be placed on what connects us to ourselves and reality, as opposed to what separates us from it. As Ratcliffe insightfully notes: “talk of feeling detached from body and world might best express an all-pervasive feeling of estrangement but, importantly, that feeling is *itself* a way of experiencing the body-world relationship and so one has not actually escaped from body and world at all” (2008:131). We must thus use this fundamental openness to the world as a powerful tool to repair the ‘lost’ connectedness to oneself. For example, by training people to repair and adjust the overweighted balance towards the inner mentalistic self, by actively and dynamically engaging with their close sensory environment via their bodily self.

This observation is supported by self-reports from DPD individuals indicating that their dissociative experiences usually trigger distressing existential questions about the nature of their ‘self’, of the reality and the meaning of the existence itself. This existential questioning is, in most of the cases, overwhelming, and impede the individual to simply ‘be there’ and enjoy life and experiences directly, as they unfold. As a recovering DPD patient strikingly expresses it:

“It came the moment where I realised that I was fully inhabiting every moment of my life, and that I couldn’t induce a feeling of depersonalisation if I tried. That was a moment of such indescribable joy, and it’s a memory that I try to hang on to when things get tough. I remember sitting at my tiny kitchen table in my studio flat, and not feeling the need to achieve or function or engage. I sat at the kitchen table for over an hour, just being. Just living” (Ciaunica & Charlton, 2018).

## Declaration of Competing Interest

The authors declare that they have no known competing financial interests or personal relationships that could have appeared to influence the work reported in this paper.

## Footnotes

1 The other major classificatory system used in contemporary psychiatry is the ICD-10 (International Classification of Diseases, World Health Organization). While there are some important differences between DSM and ICD (e.g., the latter list DPD under neurotic disorders), both they largely agree upon the diagnostic criteria for DPD, which are the following: a) persistent symptoms of DP/DR not occurring as part of another disorder or be directly substance-induced; b) the individual should not be suffering from psychosis (which would imply a different diagnosis, such as schizophrenia). DSM adds the criterion c) there should be significant distress and/or functional impairment. This seems appropriate, as otherwise it is hard to argue that the phenomena can usefully be seen as pathological (Medford et al., 2005).

2 Homeostasis is defined as ‘‘the regulation by an organism of the chemical composition of its body fluids and other aspects of its internal environment so that physiological processes can proceed at optimum rates. It involves monitoring changes in the external and internal environments by means of receptors and adjusting the composition of the body fluids accordingly; excretion and (osmotic) regulation are important in this process’’ (Medford, 2000).

3 At higher levels of the somatosensory hierarchy, it is not clear whether an attention or an enhancement of ascending sensory information may be at work. It has been proposed that these higher-level regions are the best candidates for an implementation of action-dependent weighting of self-generated versus externally caused somatosensory components (e.g., Edwards, Adams, Brown, Parees, & Friston, 2012; Paton et al., 2014).

4 For example, representationalists argue that while we typically have access only the representation’s intentional content (something in the world which it’s about) without noticing its non-intentional carrier properties (Northoff & Panksepp, 1903; Ullman et al., 1999), the process itself of constructing inner representations can become available to our introspective attention. Whenever we consciously direct our attention introspectively inwards, so to speak, the transparent processing of mental representations (typically taken for granted and hence “invisible”) becomes “*opaque*”, that is, “visible” and available to our attention (cf. the window metaphor described above).

5 The terms ‘prior beliefs’, ‘expectations’, ‘predictions’ are used here interchangeably.

6 There is also debate over the question whether a loss of prior precision (e.g., prefrontal hypoconnectivity) and gain in sensory precision (e.g., sensory hyperconnectivity) may indeed be two separate factors in the illness (see Thompson, 2018).
